# Concurrent presence of high serum uric acid and inflammation is associated with increased incidence of type 2 diabetes mellitus in Korean adult population

**DOI:** 10.1038/s41598-022-15176-9

**Published:** 2022-06-29

**Authors:** Kyung Won Lee, Dayeon Shin

**Affiliations:** 1 Department of Home Economics Education, Korea National University of Education, Cheongju, 28173 Republic of Korea; 2 Department of Food and Nutrition, Inha University, 100 Inha-ro, Michuhol-gu, Incheon, 22212 Republic of Korea

**Keywords:** Metabolic disorders, Risk factors, Epidemiology

## Abstract

Although serum uric acid level and systemic inflammation have been highlighted as risk factors for type 2 diabetes mellitus (T2DM), little is known about these associations in the Korean population. Thus, we examined the individual and combined associations of serum uric acid and systemic inflammation (evaluated using high-sensitivity C-reactive protein [hs-CRP] measurement) with the future risk of T2DM. A total of 4152 Korean adults aged 45–76 years without T2DM, cancer, or gout at baseline in 2007–2008 from the Korean Genome and Epidemiology Study were followed up until 2016. Cox proportional hazard models were used to estimate the multivariable-adjusted hazard ratios (HRs) and 95% confidence intervals (CIs) of T2DM according to sex-specific tertiles of serum uric acid and hs-CRP levels after adjustment for confounders. During the mean follow-up of 7.3 years, 548 participants developed T2DM. High serum uric acid and hs-CRP levels were independently associated with an increased incidence of T2DM. The multivariable-adjusted HRs (95% CIs) for the incidence of T2DM in the highest tertiles of serum uric acid and hs-CRP were 1.54 (1.24–1.93) and 1.90 (1.48–2.43), respectively. High levels of serum uric acid and hs-CRP in combination were associated with an increased incidence of T2DM (HR: 4.69; 95% CI: 2.81–7.84) compared to low levels of serum uric acid and hs-CRP. These findings suggest that the combination of high serum uric acid and hs-CRP levels was significantly associated with an elevated incidence of T2DM; however, their synergistic effects were not observed in middle-aged and elderly Korean adults.

## Introduction

With an increase in the burden of morbidity and premature mortality due to type 2 diabetes mellitus (T2DM), T2DM has become one of the greatest public health challenges^1^. In 2017, the global prevalence of T2DM was 8.8% (425 million adults) among adults aged 20–79 years, and it is expected to increase to 9.9% (629 million adults) by 2045^2^. Various complications resulting from T2DM, such as kidney diseases, retinopathy, foot damage, neuropathy, and cardiovascular diseases, are also significant problems^3^; thus, prevention and proper management of T2DM, as well as the identification of effective biomarkers as early predictors of T2DM, are high-priority health issues.

C-reactive protein (CRP) is an acute reactant phase protein produced in the liver. The CRP level in the blood rapidly increases in response to inflammation; therefore, CRP is commonly used as a marker of systemic inflammation^4^. Previous epidemiological studies found an association between the inflammation state (defined by CRP levels) and the risk of T2DM^5^, and that CRP concentrations varied as to ethnicity^6^. In addition to CRP, previous studies have reported that serum uric acid, which is the end-product of purine catabolism in humans^7^, is positively associated with the risk of T2DM^8–10^. Although the potential mechanisms of serum uric acid-induced T2DM remain unclear, elevated serum uric acid levels have been linked to endothelial dysfunction and oxidative stress, which in turn leads to insulin resistance and T2DM^11,12^.

Previous cohort studies have indicated that high serum uric acid levels are positively associated with the levels of CRP and inflammatory cytokines, such as interleukin (IL)-1b, IL-6, and tumor necrosis factor (TNF)-α^11,13^. Additionally, it has been hypothesized that serum uric acid may induce metabolic abnormalities through inflammatory pathways^14^. In addition, inflammatory cytokines increase the expression and activity of xanthine oxidase, an enzyme that catalyzes the conversion of uric acid precursors into uric acid^15^. Therefore, we investigated the independent and interactive effects of serum uric acid level and inflammation on metabolic dysfunction. Although a few studies have evaluated the contributory effects of uric acid and inflammation on metabolic disorders, they were limited to cross-sectional and longitudinal studies with small sample sizes or short follow-up periods. Additional evidence based on prospective cohort studies with longer follow-up periods are needed to determine whether serum uric acid or inflammation is prospectively associated with T2DM risk and whether the concurrent presence of these two factors interacts synergistically to develop T2DM.

This study aimed to explore the independent role of uric acid and systemic inflammation (measured by high-sensitivity CRP [hs-CRP] levels) in predicting the future risk of T2DM. We also prospectively investigated the combined effects of serum uric acid and hs-CRP levels on the incidence of T2DM in a population-based cohort of middle-aged and elderly Korean adults.

## Results

### Baseline characteristics

During a mean follow-up period of 7.3 ± 1.6 years (30,365 total person-years), 548 new cases of T2DM were identified. The main characteristics of the study participants at baseline across the sex-specific tertiles of serum uric acid and hs-CRP levels are summarized in Table 1. Area of residence, education level, regular physical activity, body mass index (BMI), waist circumference (WC), diastolic blood pressure, triglycerides, total cholesterol, high-density lipoprotein (HDL)-cholesterol, and fasting blood glucose at baseline significantly differed according to tertiles of serum uric acid and hs-CRP levels (all, *P* < 0.05). Participants in the highest tertile of serum uric acid levels (serum uric acid ranges 6.2 to 11.8 mg/dL for men; 4.5 to 10.6 mg/dL for women) were more likely to live in Ansung (rural area), drink more alcohol, exercise regularly, and have high BMI, WC, diastolic blood pressure, triglycerides, total cholesterol and fasting blood glucose levels and low HDL-cholesterol levels. Participants with the highest tertile of hs-CRP levels (hs-CRP ranges 1.05 to 9.82 mg/L for men; 0.90 to 9.88 mg/L for women) tended to be older, urban area (Ansan) residents, less educated, current smokers, less physically active, and have high BMI, WC, systolic and diastolic blood pressure, triglycerides, total cholesterol, and fasting blood glucose levels. Those from the highest tertile of serum uric acid or hs-CRP levels were more likely to have low HDL-cholesterol levels.

**Table 1:** Baseline characteristics of the study participants by tertile of serum uric acid and high-sensitivity C-reactive protein levels in Korean adults aged 45–76 years.

	Tertile of uric acid^1^			*P*	Tertile of hs-CRP^2^			*P*
	T1 (lowest) (n = 1382)	T2 (n = 1362)	T3 (highest) (n = 1408)		T1 (lowest) (n = 1382)	T2 (n = 1383)	T3 (highest) (n = 1387)	
Sex				0.4301				0.9010
Men	612 (44.3)^3^	660 (48.5)	603 (42.8)		628 (45.4)	620 (44.8)	627 (45.2)	
Women	770 (55.7)	702 (51.5)	805 (57.2)		754 (54.6)	763 (55.2)	760 (54.8)	
Age (years)	57.2 ± 8.6	56.6 ± 8.3	57.1 ± 8.4	0.0759	55.5 ± 8.1	57.31 ± 8.4	58.07 ± 8.6	< 0.0001
Area of residence				< 0.0001				0.0176
Ansan	786 (56.9)	675 (49.6)	682 (48.4)		686 (49.6)	706 (51.1)	751 (54.2)	
Ansung	596 (43.1)	687 (50.4)	726 (51.6)		696 (50.4)	677 (49.0)	636 (45.9)	
Education level				0.0296				< 0.0001
≤ elementary school	450 (32.6)	367 (26.9)	429 (30.5)		349 (25.3)	440 (31.8)	457 (33.0)	
Middle/high school	769 (55.6)	783 (57.5)	767 (54.5)		824 (59.6)	741 (53.6)	754 (54.4)	
≥ college	163 (11.8)	212 (15.6)	212 (15.0)		209 (15.1)	202 (14.6)	176 (12.7)	
Smoking status				0.8038				0.0006
Never	927 (67.1)	872 (64.0)	938 (66.6)		947 (68.5)	902 (65.2)	888 (64.0)	
Past	244 (17.6)	249 (18.3)	251 (17.8)		255 (18.5)	245 (17.7)	244 (17.6)	
Current	211 (15.3)	241 (17.7)	219 (15.6)		180 (13.0)	236 (17.1)	255 (18.4)	
Alcohol consumption (grams/day)	6.67 ± 16.7	8.2 ± 17.5	9.6 ± 20.7	0.0001	7.78 ± 17.5	8.5 ± 19.3	8.3 ± 18.5	0.6037
Regular physical activity				0.0178				0.0080
Yes	506 (36.6)	522 (38.3)	577 (41.0)		567 (41.0)	537 (38.8)	501 (36.1)	
No	876 (63.4)	840 (61.7)	831 (59.0)		815 (59.0)	846 (61.2)	886 (63.9)	
Body mass index (kg/m^2^)	23.6 ± 2.9	24.3 ± 2.9	25.2 ± 3.0	< 0.0001	23.4 ± 2.6	24.5 ± 2.7	25.2 ± 3.3	< 0.0001
Waist circumference (cm)	81.9 ± 9.2	83.4 ± 9.4	85.6 ± 9.7	< 0.0001	80.6 ± 8.8	83.9 ± 9.2	86.5 ± 9.8	< 0.0001
Systolic blood pressure (mmHg)	116.7 ± 16.4	116.9 ± 16.0	117.9 ± 16.1	0.0964	114.9 ± 15.6	117.5 ± 16.0	119.2 ± 16.7	< 0.0001
Diastolic blood pressure (mmHg)	76.1 ± 9.5	77.1 ± 9.7	78.3 ± 9.9	< 0.0001	75.9 ± 9.5	77.3 ± 9.9	78.2 ± 9.6	< 0.0001
Triglycerides (mg/dL)	132.9 ± 74.6	148.2 ± 97.8	168.9 ± 97.9	< 0.0001	133.3 ± 72.2	151.5 ± 87.7	165.6 ± 10.9.3	< 0.0001
Total cholesterol (mg/dL)	183.7 ± 32.6	188.9 ± 33.6	193.8 ± 35.0	< 0.0001	184.4 ± 32.3	190.1 ± 34.2	192.0 ± 35.0	< 0.0001
HDL-cholesterol (mg/dL)	45.9 ± 9.9	44.9 ± 9.8	44.2 ± 9.8	< 0.0001	46.0 ± 10.1	45.2 ± 10.0	43.8 ± 9.5	< 0.0001
Fasting blood glucose (mg/dL)	90.7 ± 8.2	91.4 ± 8.9	92.3 ± 9.1	< 0.0001	90.4 ± 8.4	91.5 ± 8.8	92.5 ± 9.1	< 0.0001
Family history of diabetes				0.3569				0.3148
Yes	22 (1.6)	25 (1.8)	29 (2.1)		27 (2.0)	29 (2.1)	20 (1.4)	
No	1,360 (98.4)	1,337 (98.2)	1,379 (97.9)		1,355 (98.1)	1,354 (97.9)	1,367 (98.6)	

*T* tertile; *hs-CRP* high-sensitivity C-reactive protein; *HDL-cholesterol* high-density lipoprotein cholesterol.

^1^Cut-offs for tertiles 1–3 of serum uric acid levels are as follows: < 5.2, 5.2–6.1, and > 6.1 mg/dL in men and < 3.8, 3.8–4.4, and > 4.4 mg/dL in women, respectively.

^2^Cut-offs for tertiles 1–3 of hs-CRP levels are as follows: < 0.45, 0.45–1.04, and > 1.04 mg/L in men and < 0.38, 0.38–0.89, and > 0.89 mg/L in women, respectively.

^3^Values are number (percentage) for categorical variables and mean ± standard deviation for continuous variables.

### Independent associations of serum uric acid and hs-CRP on incident T2DM risk

The incidence rates, hazard ratios (HRs) and 95% confidence intervals (CIs) for T2DM are provided according to tertiles, continuous measures, and dichotomous categories of serum uric acid and hs-CRP levels in Tables 2 and 3. Participants in the highest tertile of serum uric acid had a 54% increased risk of T2DM compared to those in the lowest tertile after controlling for potential confounders (fully adjusted HR: 1.54; 95% CI: 1.24–1.93; *P*_trend_ < 0.0001) (Table 2). Moreover, the fully adjusted HR was 1.47 (95% CI: 1.02–2.12) per 1 log mg/dL increase in serum uric acid levels. However, when we dichotomized serum uric acid levels (hyperuricemia vs. non-hyperuricemia), the associations were not significant (fully adjusted HR: 1.12; 95% CI: 0.87–1.44).

**Table 2:** Adjusted hazard ratios (with 95% confidence intervals) for type 2 diabetes according to serum uric acid levels in Korean adults aged 45–76 years.

	Tertile of serum uric acid^1^			*P*_trend_	Per 1 log unit (mg/dL) incline in serum uric acid	Hyperuricemia (vs. non-hyperuricemia)^2^
	T1 (lowest)	T2	T3 (highest)			
	HR (95% CI)	HR (95% CI)	HR (95% CI)		HR (95% CI)	HR (95% CI)
Total (n = 4152)						
Person-years	10,282	10,060	10,023		30,365	2699
Incident cases (*n*)	127	152	269		548	81
Rate per 1000 person-years	12.4	15.1	26.8		18.0	30.0
Model 1^3^	1.00	1.25 (0.99–1.59)	2.22 (1.80–2.74)	< 0.0001	3.15 (2.19–4.53)	1.82 (1.43–2.31)
Model 2	1.00	1.07 (0.84–1.36)	1.63 (1.31–2.02)	< 0.0001	1.62 (1.13–2.34)	1.18 (0.92–1.51)
Model 3	1.00	1.05 (0.83–1.33)	1.54 (1.24–1.93)	< 0.0001	1.47 (1.02–2.12)	1.12 (0.87–1.44)

*T* tertile; *HR* hazard ratio; *CI* confidence interval.

^1^Cut-offs for tertiles 1–3 of serum uric acid levels are as follows: < 5.2, 5.2–6.1, and > 6.1 mg/dL in men and < 3.8, 3.8–4.4, and > 4.4 mg/dL in women, respectively.

^2^Hyperuricemia is defined as serum uric acid levels ≥ 7 mg/dL in men and ≥ 6 mg/dL in women.

^3^Model 1: adjusted for sex (men or women) and age (years); Model 2: additionally adjusted for area of residence (Ansan or Ansung), education level (≤ elementary school, middle/high school, or ≥ college), smoking status (never, past, or current), alcohol consumption (g/d), regular physical activity (yes or no), body mass index (kg/m^2^), waist circumference (cm), systolic and diastolic blood pressure (mmHg), triglyceride (mg/dL), total cholesterol (mg/dL), high-density lipoprotein cholesterol (mg/dL) and fasting blood glucose level (mg/dL), and family history of diabetes (yes or no); Model 3: additionally adjusted for log-transformed hs-CRP levels.

**Table 3:** Adjusted hazard ratios (with 95% confidence intervals) for type 2 diabetes according to high-sensitivity C-reactive protein levels in Korean adults aged 45–76 years.

	Tertile of hs-CRP^1^			*P*_trend_	Per 1 log unit (mg/L) incline in hs-CRP	High inflammation^2^ (vs. low inflammation)
	T1 (lowest)	T2	T3 (highest)			
	HR (95% CI)	HR (95% CI)	HR (95% CI)		HR (95% CI)	HR (95% CI)
Total (n = 4152)						
P erson-years	10,401	10,079	9,885		30,365	3,760
Incident cases (*n*)	98	184	255		548	109
Rate per 1000 person-years	9.4	18.3	25.8		18.0	29.0
Model 1^3^	1.00	1.81 (1.41–2.31)	2.66 (2.11–3.36)	< 0.0001	1.38 (1.27–1.50)	1.68 (1.36–2.08)
Model 2	1.00	1.51 (1.17–1.94)	1.96 (1.53–2.50)	< 0.0001	1.19 (1.09–1.30)	1.30 (1.05–1.61)
Model 3	1.00	1.48 (1.15–1.91)	1.90 (1.48–2.43)	< 0.0001	1.17 (1.07–1.29)	1.27 (1.02–1.57)

*T* tertile; *hs-CRP* high-sensitivity C-reactive protein; *HR* hazard ratio; *CI* confidence interval.

^1^Cut-offs for tertiles 1–3 of hs-CRP levels are as follows: < 0.45, 0.45–1.04, and > 1.04 mg/L in men and < 0.38, 0.38–0.89, and > 0.89 mg/L in women, respectively.

^2^High inflammation is defined as > 2 mg/L of hs-CRP levels.

^3^Model 1: adjusted for sex (men or women) and age (years); Model 2: additionally adjusted for area of residence (Ansan or Ansung), education level (≤ elementary school, middle/high school, or ≥ college), smoking status (never, past, or current), alcohol consumption (g/d), regular physical activity (yes or no), body mass index (kg/m^2^), waist circumference (cm), systolic and diastolic blood pressure (mmHg), triglyceride (mg/dL), total cholesterol (mg/dL), high-density lipoprotein cholesterol (mg/dL) and fasting blood glucose level (mg/dL), and family history of diabetes (yes or no); Model 3: additionally adjusted for log-transformed serum uric acid levels.

In the fully adjusted models, participants in the highest tertile of hs-CRP levels had an 90% increase in the risk of T2DM (fully adjusted HR: 1.90; 95% CI: 1.48–2.43; *P*_trend_ < 0.0001) (Table 3). On a continuous basis, hs-CRP levels were also significantly associated with an increased incidence of T2DM (fully adjusted HR per 1 log mg/L increase: 1.17; 95% CI: 1.07–1.29). Similarly, participants with high inflammation (> 2 mg/L of hs-CRP) showed a 27% increased risk of T2DM (fully adjusted HR: 1.27; 95% CI: 1.02–1.57) than those with low inflammation (≤ 2 mg/L of hs-CRP).

### Combined associations of serum uric acid and hs-CRP on incident T2DM

The associations between serum uric acid and hs-CRP levels and the incidence of T2DM are shown in Table 4. Although the test for the multiplicative interaction between serum uric acid and hs-CRP levels was not statistically significant (*P*_interaction_ = 0.3790), when cross-classifying participants by both exposure variables, the risk of developing T2DM was the highest among participants with the combination of the highest tertile of serum uric acid and hs-CRP levels (HR: 4.69; 95% CI: 2.81–7.84) compared with the opposite extreme.

**Table 4:** Association of type 2 diabetes by serum uric acid and high-sensitivity C-reactive protein level strata in Korean adults aged 45–76 years.

		Tertile of serum uric acid^1^		
		T1 (lowest)	T2	T3 (highest)
		HR (95% CI)^3^	HR (95% CI)	HR (95% CI)
Tertile of hs-CRP^2^	T1 (lowest)	1.00	1.49 (0.87–2.55)	2.79 (1.58–4.94)**
	T2	1.90 (1.21–2.98)**	2.00 (1.24–3.21)**	3.69 (2.17–6.25)**
	T3 (highest)	1.89 (1.20–2.98)**	2.79 (1.75–4.46)**	4.69 (2.81–7.84)**

*T* tertile; *hs-CRP* high-sensitivity C-reactive protein; *HR* hazard ratio; *CI* confidence interval.

^1^Cut-offs for tertiles 1–3 of serum uric acid levels are as follows: < 5.2, 5.2–6.1, and > 6.1 mg/dL in men and < 3.8, 3.8–4.4, and > 4.4 mg/dL in women, respectively.

^2^Cut-offs for tertiles 1–3 of hs-CRP levels are as follows: < 0.45, 0.45–1.04, and > 1.04 mg/L in men and < 0.38, 0.38–0.89, and > 0.89 mg/L in women, respectively.

^3^Individuals in the lowest tertile of serum uric acid and hs-CRP levels are used as the reference group. The model is adjusted for sex (men or women), age (years), area of residence (Ansan or Ansung), education level (≤ elementary school, middle/high school, or ≥ college), smoking status (never, past, or current), alcohol consumption (g/d), regular physical activity (yes or no), body mass index (kg/m^2^), waist circumference (cm), systolic and diastolic blood pressure (mmHg), triglyceride (mg/dL), total cholesterol (mg/dL), high-density lipoprotein cholesterol (mg/dL) and fasting blood glucose level (mg/dL), and family history of diabetes (yes or no). ** *P* < 0.001.

### Predicting incident T2DM with serum uric acid and hs-CRP

A comparison of the performance of each model in predicting T2DM is presented in Table 5. The model with both serum uric acid and hs-CRP levels showed the best predictive accuracy, with an area under the receiver operating characteristic curve (AUC) between 0.731 and 0.930, and the highest Uno’s C-statistic (0.758) during the follow-up periods. C-statistics of the prediction models including at least one of the markers of serum uric acid and hs-CRP were significantly higher than those of the conventional model (all, *P* < 0.0001). The predictive model including both serum uric acid and hs-CRP levels provided a better C-statistic than the model with only serum uric acid levels (*P* = 0.0030); however, there was no significant difference in the model with only hs-CRP levels (*P* = 0.2445).

**Table 5:** Overall ability of models predicting type 2 diabetes incidence in Korean adults aged 45–76 years.

Model	AUC for the incidence of T2DM				Differentiate incidence		
	1-year	5-year	9-year	C-statistic^2^	*P*^3^	*P*^4^	*P*^5^
Conventional model^1^	0.766	0.635	0.691	0.665	Reference	–	–
+ serum uric acid	0.921	0.720	0.747	0.748	< 0.0001	Reference	–
+ hs-CRP	0.913	0.726	0.749	0.753	< 0.0001	0.1366	Reference
+ serum uric acid and hs-CRP	0.930	0.731	0.754	0.758	< 0.0001	0.0030	0.2445

*AUC* area under the receiver operating characteristic curve; *hs-CRP* high-sensitivity C-reactive protein; T2DM, type 2 diabetes mellitus.

^1^The conventional model is adjusted for sex (men or women), age (years), area of residence (Ansan or Ansung), education level (≤ elementary school, middle/high school, or ≥ college), smoking status (never, past, or current), alcohol consumption (g/day), regular physical activity (yes or no), body mass index (kg/m^2^), waist circumference (cm), systolic and diastolic blood pressure (mmHg), triglyceride (mg/dL), total cholesterol (mg/dL), high-density lipoprotein cholesterol (mg/dL) and fasting blood glucose level (mg/dL), and family history of diabetes (yes or no).

^2^Uno’s C-statistic from Cox linear models.

^3^Compared with C-statistic of conventional model.

^4^Compared with C-statistic of + serum uric acid model.

^5^Compared with C-statistic of + hs-CRP model.

## Discussion

Our prospective cohort study of middle-aged and elderly Korean adults (mean follow-up time of 7.3 years) showed that elevated serum uric acid and hs-CRP levels increased the risk of T2DM development. For each 1 log unit increase in serum uric acid (mg/dL) and hs-CRP (mg/L), the incidence of T2DM increased by 47% and 17%, respectively. These associations were independent of conventional T2DM risk factors such as sex, age, residential area, education level, smoking status, alcohol consumption, physical activity, BMI, WC, blood pressure, serum lipid and fasting blood glucose levels, and family history of diabetes. Furthermore, high levels of serum uric acid, in combination with hs-CRP, exacerbated the future risk of developing T2DM, but these two factors did not act synergistically.

To the best of our knowledge, this is the first population-based cohort study to investigate the combined effects of serum uric acid and hs-CRP levels on the incidence of T2DM from a longitudinal perspective. Previous studies conducted in the United States and Europe have demonstrated positive associations between serum uric acid levels and the risk of T2DM. Data from the Framingham Heart Study^9^ and Rotterdam Study^8^ showed that elevated serum uric acid levels were significantly associated with an increased risk of T2DM. However, the role of serum uric acid level in T2DM development in Asian populations has not been consistent. A prospective study of Japanese men reported no association between serum uric acid levels and the incidence of T2DM^16^. In contrast, a cohort study of 2,690 Chinese adults aged 35–97 years showed that the risk of T2DM was higher in individuals with the highest levels of serum uric acid than in those with the lowest levels (relative risk for Q5 vs. Q1: 1.40; 95% CI: 1.02–1.92; *P*_trend_ < 0.05)^17^. Along with a previous Chinese study, the present study found significant positive associations between serum uric acid levels and the incidence of T2DM, regardless of how we defined the exposure variable in the analytic models. Our findings reflect the robustness of these associations and suggest the potential role of elevated serum uric acid levels as an independent risk factor for T2DM among Korean adults.

As insulin resistance and T2DM may result from subclinical chronic low-grade inflammation, inflammatory cytokine levels have been reported to serve as effective biomarkers for T2DM^18^. CRP is commonly used as a marker of systemic inflammation, and its association with T2DM has been evaluated in various non-diabetic populations. Some cross-sectional studies have shown that CRP levels vary substantially among different ethnic groups^6,19^. Kelley et al.^6^ compared CRP levels among different ethnicities and reported that CRP concentrations were the highest among African Americans (median: 3.2 mg/L), followed by Hispanics (median: 2.3 mg/L), Caucasians (median: 1.5 mg/L), Chinese (median: 0.7 mg/L), and Japanese (median: 0.5 mg/L). In our study, the median hs-CRP level in Koreans was 0.6 mg/L, which was similar to than in other Asian groups and much lower than that in Westerners. Although differences in CRP distributions across populations have been acknowledged, previous analyses of cohort studies from various populations indicated relatively consistent findings, suggesting a positive association between CRP levels and the incidence of T2DM. A recent meta-analysis of 19 prospective studies conducted predominantly in Western countries showed a 25% increase in the risk of T2DM per 1 log pg/mL increment in CRP concentrations^5^. In agreement with our results, previous studies with Asian populations, in which relatively low CRP levels were observed, also reported positive associations between CRP levels and the risk of T2DM development^20,21^. Moreover, although we presented the prospective associations of hs-CRP levels with an increased risk of T2DM among Korean adults, it is limited to determining Korean-specific CRP cut-off values for predicting the future risk of T2DM. Therefore, further studies are needed to establish the optimal hs-CRP cut-off points that could identify high-risk groups for T2DM development among Koreans.

Several studies have shown a significant relationship between serum uric acid concentrations and the levels of CRP and pro-inflammatory markers involved in the causal pathways of T2DM. In observational studies, serum uric acid levels were positively associated with CRP levels^22,23^, TNF-α^23^, and IL-6^13^. However, the underlying mechanisms by which uric acid increases the levels of inflammatory markers remain unclear. One possible explanation is that damaged cells and tissues release uric acid, which triggers inflammatory cytokines^24^. Results from an experimental study with human vascular and endothelial cells support the hypothesis that high serum uric acid concentrations stimulate CRP expression^11^. In contrast, pro-inflammatory cytokines play an important role in uric acid metabolism by upregulating the expression of xanthine oxidase^14^, which plays a key role in uric acid metabolism by converting xanthine to uric acid^25^. The increased expression of xanthine oxidase promotes the production of uric acid and superoxide free radicals^15^, resulting in endothelial dysfunction that may cause T2DM^12^. Therefore, studies exploring the role of the vicious cycle between uric acid and inflammation in the onset of metabolic disorders, including T2DM, are needed. However, to date, although the separate effects of serum uric acid and hs-CRP levels on the risk of T2DM have been recognized^26,27^, the combined effect of these two biomarkers on T2DM has not been investigated. Only a few cross-sectional studies have shown that the concurrent presence of elevated uric acid and CRP levels is associated with the risk of hypertension^28^, metabolic syndrome, and insulin resistance^29^. In our study, findings from the large cohort with long-term follow-up may provide new insights into the prospective relationships of coexisting elevated serum uric acid and hs-CRP levels with the incidence of T2DM, even though their effects were not synergistic. In addition, we estimated the C-statistics and AUCs to evaluate the usefulness of serum uric acid with concomitant hs-CRP levels in predicting the future risk of T2DM. Our findings imply that identifying concurrent elevated serum uric acid and hs-CRP levels as T2DM risk factors may expand opportunities for the early detection and prevention of T2DM.

The strengths of this study include its prospective design, relatively long follow-up period, and the inclusion of detailed information on potential confounders. Furthermore, this study extended the findings of previous studies conducted in Western populations and provided additional evidence of the separate and combined contributions of serum uric acid and hs-CRP levels to the incidence of T2DM among populations with relatively low hs-CRP levels. This study is not without potential limitations. First, the study population was composed of middle-aged and older adults residing in specific regions of Korea, which may limit the generalizability of the findings to the entire Korean population. However, the distributions of baseline serum uric acid and hs-CRP levels in our study population did not vary significantly from those of previous nationwide or cohort studies^30,31^; Thus, the associations observed in this study might be similar for other Korean populations. Second, dietary data were not available at baseline. However, we could evaluate the effect of alcohol consumption, which is a major dietary factor affecting serum uric acid levels, in the analytical models. Moreover, even after adjusting the analytical models for confounders related to T2DM, significant associations between serum uric acid and hs-CRP levels and the incidence of T2DM were still maintained. Third, all analyses were based on single measurements of serum uric acid and hs-CRP levels, which limited the conclusions regarding the effects of changes in serum uric acid and inflammation levels on T2DM development over time. Future longitudinal studies that include repeated measurements of serum uric acid and hs-CRP levels and incorporate dietary information are warranted.

## Conclusions

In summary, elevated levels of serum uric acid and hs-CRP were independently associated with an increased incidence of T2DM. Although there was no significant synergistic effect of these two indicators, the concurrent presence of elevated serum uric acid and hs-CRP levels may exacerbate the risk of T2DM development in a large Korean cohort aged 45 years and older. Our findings suggest that monitoring serum uric acid and hs-CRP levels can be an effective strategy for identifying individuals at high risk of T2DM development among Korean adults.

### Research design and methods

#### Data source and study population

**Figure 1:**
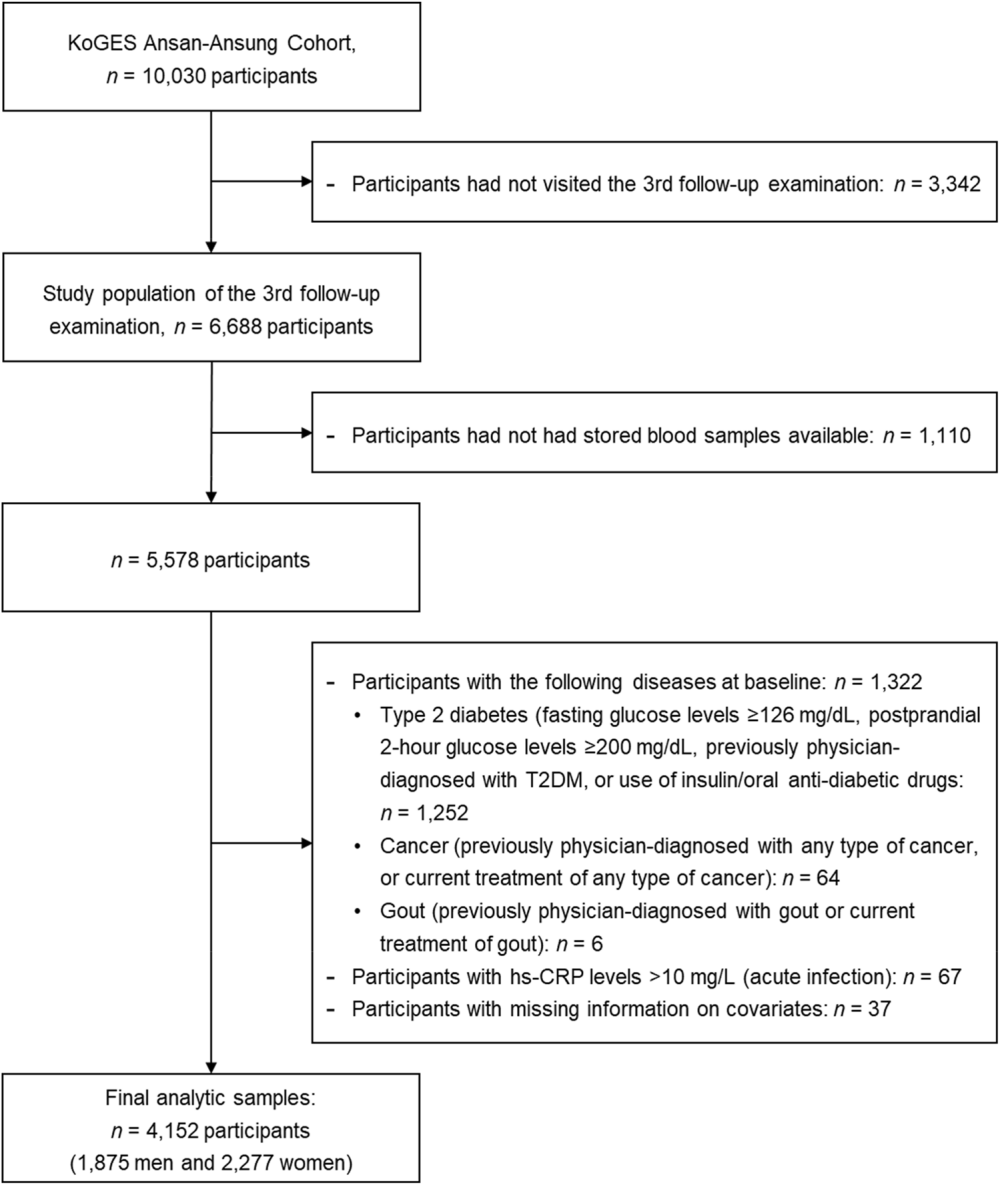
Flowchart of the study population.

We used data from the Ansan–Ansung cohort study of the Korean Genome and Epidemiology Study (KoGES). In two Korean cities (Ansan and Ansung), 10,030 Korean adults aged between 40 and 69 years were recruited between 2001 and 2002 and followed up biennially. As previously described, data on sociodemographics, lifestyle, medical and medication history, and reproductive health were collected. Health examinations and blood and urine tests were performed by trained staff and interviewers using standardized protocols at baseline and subsequent follow-up examinations^32^. In 2018, the Korea Center for Disease Control and Prevention (KCDC) newly released data on the levels of 15 biomarkers, including serum uric acid, from stored serum samples collected at the 3^rd^ follow-up examination between 2007 and 2008. Therefore, in this study, data from the 3rd follow-up examination were considered as the baseline to include data on serum uric acid levels. From a total of 6688 participants who completed the 3^rd^ follow-up examination (2007–2008), 1,110 were excluded because stored serum samples were not available. We further excluded participants with T2DM (n = 1252), any type of cancer (n = 64), or gout (n = 6). Of these, 37 participants were not suitable for the current study because of missing information on covariates. To rule out confounding by acute infection, participants with hs-CRP levels of > 10 mg/L were excluded from the final analysis (n = 67). Finally, 4152 Korean adults (1875 men and 2277 women) who were followed-up until 2016 were included in this study (Fig. 1). The KoGES study was reviewed and approved by the Institutional Review Board of the Korea Centers for Disease Control and Prevention, and written informed consent was obtained from all the participants. All methods were performed in compliance with relevant institutional guidelines and regulations. The protocol was reviewed and approved by the Institutional Review Board of Korea National University of Education on November 24, 2020 (IRB No. KNUE-202011-BMBR-0102-01).

#### Assessment of serum uric acid and hs-CRP

Blood samples for the laboratory tests were collected after overnight fasting. Serum uric acid and hs-CRP concentrations were assessed using enzymatic colorimetric methods with an automatic analyzer (ADVIA 1650 and 1800, Siemens, Tarrytown, NY, USA). Serum uric acid and hs-CRP levels, the main exposure variables of interest, were analyzed as tertiles based on sex-specific distributions and as continuous measures. Both exposure variables were analyzed as dichotomous variables. For dichotomous analyses, hyperuricemia was defined as serum uric acid levels ≥ 7 mg/dL in men and ≥ 6 mg/dL in women^33^. This is the saturation point of monosodium urate, which is the prevalent form of uric acid in extracellular fluids^34^. High inflammation was defined as hs-CRP levels > 2 mg/L in both men and women, as previously used in the Asian population^35^.

### Ascertainment of T2DM

The incidence of T2DM was determined if participants had fasting blood glucose levels ≥ 126 mg/dL or 2-h oral glucose tolerance tests (OGTT) ≥ 200 mg/dL at follow-up examinations, based on the World Health Organization^36^ and American Diabetes Association^37^. In addition, participants who were newly diagnosed with T2DM after the previous examination or who used insulin or oral anti-diabetic medicine were considered T2DM cases.

### Statistical analyses

Differences in the general characteristics of study participants across the categories of serum uric acid and hs-CRP levels were tested using the Mantel–Haenszel chi-square test for categorical variables and generalized linear regression for continuous variables, respectively. Data for serum uric acid and hs-CRP levels were right-skewed and log-transformed before the analysis.

Multivariable Cox proportional hazard models were used to estimate the future risk of T2DM development. To test for linear trends, we used the median of each tertile of serum uric acid and hs-CRP levels as continuous variables. The following covariates were used for adjustment: sex (men or women) and age (years) were adjusted in Model 1; area of residence (Ansan or Ansung), education level (≤ elementary school, middle/high school, or ≥ college), smoking status (never, past, or current), alcohol consumption (g/d), regular physical activity (yes or no), BMI (kg/m^2^), WC (cm), systolic and diastolic blood pressure (mmHg), triglyceride (mg/dL), total cholesterol (mg/dL), HDL-cholesterol (mg/dL), fasting blood glucose (mg/dL), and family history of diabetes (yes or no) were additionally adjusted in Model 2; log-transformed serum uric acid or hs-CRP levels were additionally adjusted in Model 3. To explore the combined effect of serum uric acid and hs-CRP levels on the incidence of T2DM, we cross-classified participants according to tertiles of serum uric acid and hs-CRP levels and analyzed the interactions between serum uric acid and hs-CRP levels and the incidence of T2DM. To test for potential interactions between serum uric acid and hs-CRP levels, we applied multiplicative interaction by including corresponding interaction terms (serum uric acid level × serum hs-CRP level, treated as log-transformed continuous variables) in the fully adjusted models. The lowest tertile of both variables was used as the reference.

Uno’s C-statistics from the Cox models were used to assess the differences in the overall prediction ability among the T2DM incidence predictive models. The conventional prediction model included established T2DM risk factors such as sex, age, residential area, education level, smoking status, alcohol consumption, regular physical activity, BMI, WC, systolic and diastolic blood pressure, triglyceride, total cholesterol, HDL-cholesterol, fasting glucose levels, and family history of diabetes. In the other three prediction models, serum uric acid and hs-CRP levels were additionally included individually or together, and the predictive abilities of the three models were compared with the conventional model.

SAS version 9.4 (SAS Institute, Cary, NC, USA) was used for all statistical analyses. Statistical tests were two-sided, and a *P* value < 0.05 was considered significant.
